# Enantioselective γ-borylation of unsaturated amides and stereoretentive Suzuki–Miyaura cross-coupling†
†Electronic supplementary information (ESI) available: Catalyst optimization data; experimental procedures; compound characterization data; spectra. See DOI: 10.1039/c7sc01093a



**DOI:** 10.1039/c7sc01093a

**Published:** 2017-05-03

**Authors:** Gia L. Hoang, James M. Takacs

**Affiliations:** a Department of Chemistry , University of Nebraska-Lincoln , Lincoln , NE 68588-0304 , USA . Email: jtakacs1@unl.edu

## Abstract

The rhodium-catalyzed, directed catalytic asymmetric hydroboration of γ,δ-unsaturated amides affords a direct route to chiral acyclic secondary γ-borylated carbonyl derivatives in high enantiomeric purity. In contrast to a similar β-borylated amide derivative, the γ-borylated amide undergoes Suzuki–Miyaura cross-coupling with stereoretention. The utility of the boronic ester products is further illustrated by other stereospecific C–B bond transformations leading to γ-amino acid derivatives, 1,4-amino alcohols, and 5-substituted-γ-lactone and γ-lactam ring systems.

## Abstract 2

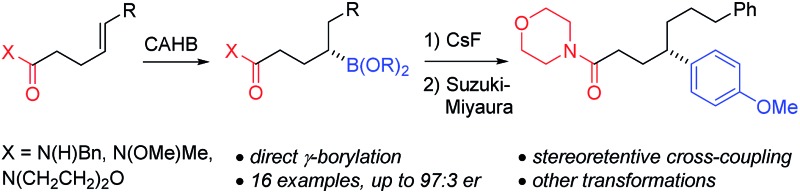
Amide-directed CAHB provides a direct route to chiral acyclic secondary γ-borylated carbonyl compounds which undergo a variety of stereospecific transformations including stereoretentive palladium-catalyzed Suzuki–Miyaura cross-coupling.

## Introduction

**Fig. 1:**
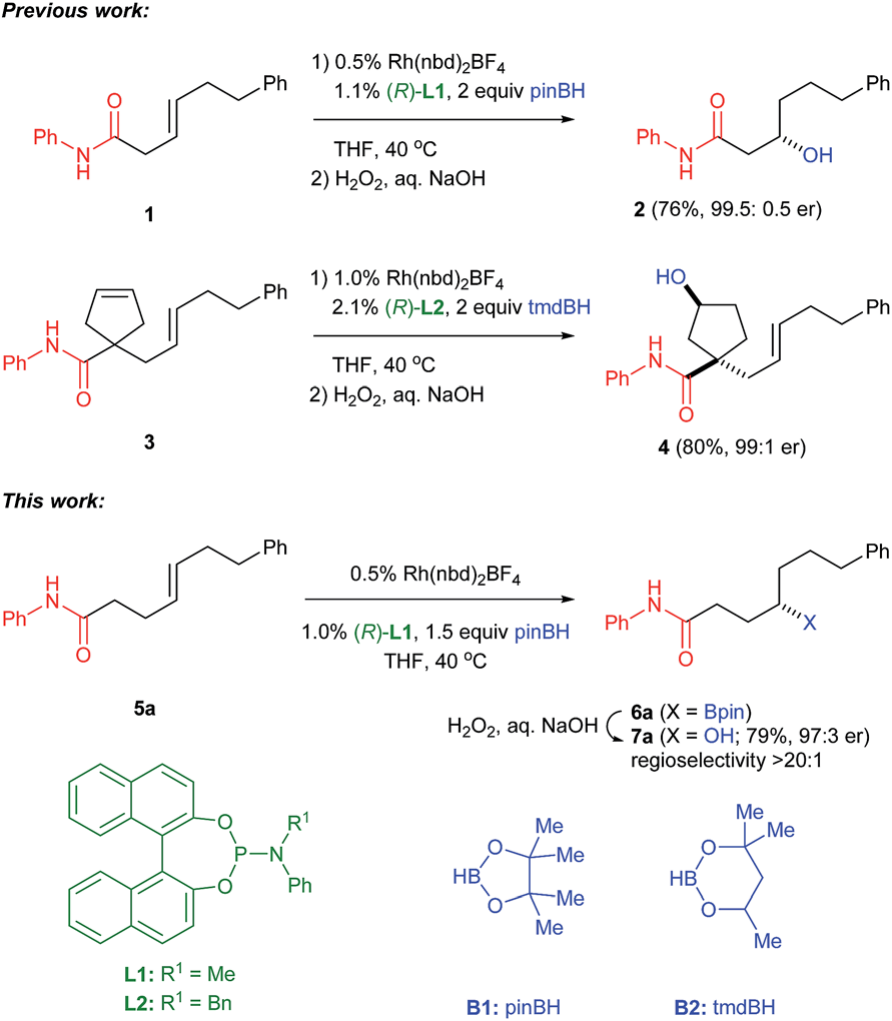
CAHB/oxidation of β,γ- *versus* γ,δ-unsaturated amides.

Chiral boronic esters are valuable synthetic intermediates for a variety of stereospecific transformations.^1–3^ Consequently, an assortment of enantioselective routes for their preparation are under active development.^4–19^ We have focused on the rhodium-catalyzed catalytic asymmetric hydroboration (CAHB) of β,γ-unsaturated amides,^20^ esters,^20*a*^ and oxime ethers^21^ for the preparation of functionalized chiral boronic esters. For example, disubstituted alkenes such as (*E*)-**1** undergo highly enantioselective β-borylation by pinacolborane (pinBH) when catalyzed by the combination of (*R*)-**L1** with [Rh(nbd)_2_BF_4_] (*i.e.*, 2 : 1 **L1** : Rh). CAHB followed by oxidation of the C–B bond affords β-hydroxy amide (*S*)-**2** in an enantiomeric ratio (er) greater than 99 : 1 (Fig. 1).^20*e*^

In an effort to expand the scope of CAHB, γ,δ-unsaturated amides such as **3** were explored.^22^ Such substrates differ from **1** in a number of important ways including, (i) the alkene is more remote than in the β,γ-unsaturated amides, (ii) substrate **3** contains two distinctly different alkene moieties, although each is ostensibly positioned γ,δ- with respect to the carbonyl directing group, and (iii) enantioselective borylation of the endocyclic alkene requires controlling the stereochemical course by desymmetrization rather than π-facial discrimination, a fundamentally different set of requirements.^23^ In the event, substrate **3** undergoes CAHB/oxidation to give the monounsaturated γ-hydroxy amide (1*S*,3*S*)-**4** in high yield (80%), regioselectivity (>20 : 1) and enantioselectivity (99 : 1 er). Only the endocyclic double bond in **3** undergoes reaction; the pendant acyclic alkene is untouched. We now report the efficient, regioselective γ-borylation of a similarly disposed alkene in γ,δ-unsaturated amide **5a** (Fig. 1). CAHB by pinBH (0.5% [Rh(nbd)_2_BF_4_/2 (*R*)-**L1**]) followed by oxidation affords chiral γ-hydroxy amide (*S*)-**7a** in 79% overall yield (97 : 3 er). While conjugate addition^11^ and C–H activation^10*a*^ methodologies provide efficient alternatives to CAHB for enantioselective β-borylation of carbonyl compounds, direct γ-borylation is unique to CAHB.

## Results and discussion

**Fig. 2:**
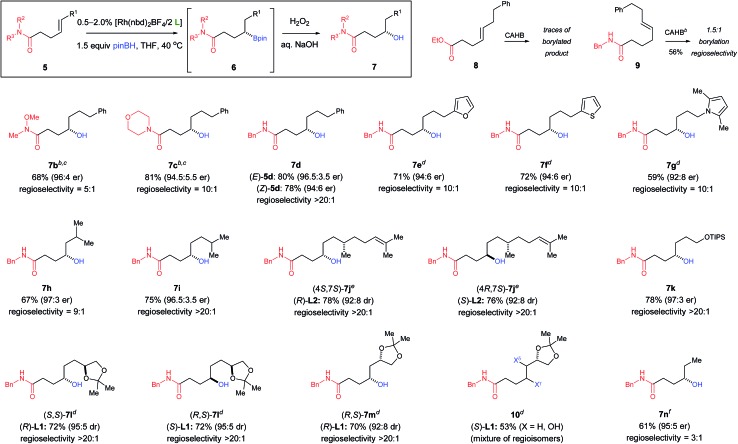
Substrate scope for CAHB of γ,δ-unsaturated amides. ^*a*^Unless otherwise noted all reaction use 0.5% [Rh(nbd)_2_BF_4_/2 (*R*)-**L1**], 1.5 equiv. pinBH, THF, 40 °C (12 h) followed by oxidation using H_2_O_2_/aq. NaOH. Unless otherwise noted, the isolated yield is that of the major regioisomer and reflects the average of three experiments generally exhibiting a spread of ±2%; regioselectivity is determined from the crude ^1^H NMR of **7**. Enantiomer ratios (er) are determined by chiral HPLC analysis; diastereomer ratios (dr) are determined for the purified mixture of diastereomers by integrating major and minor ^13^C NMR resonances. ^*b*^2.0% [Rh(nbd)_2_BF_4_/2 (*R*)-**L1**]. ^*c*^Oxidation conditions: NaBO_3_/H_2_O. ^*d*^1.0% [Rh(nbd)_2_BF_4_/2 **L1**]; (*R*)-**L1** is used unless noted otherwise in the figure. ^*e*^1.0% [Rh(nbd)_2_BF_4_/2 **L2**]. ^*f*^er is determined by ^19^F NMR of the corresponding Mosher ester (see ESI for details†).

The γ,δ-unsaturated amides (*E*)-**5b–n** shown in Fig. 2, along with several related structures, were treated with the catalyst system for γ-borylation (*i.e.*, 2 : 1 combinations of **L1** or **L2** with [Rh(nbd)_2_BF_4_], 0.5 mol% unless otherwise noted). In addition to phenyl amide **5a** described above, the corresponding Weinreb amide **5b**, the morpholine amide^24^ **5c** and benzyl amide **5d** undergo CAHB to afford the intermediate γ-borylated amides **6b–d** with high levels of enantioselectivity. Within this series of amides, the secondary amides (*i.e.*, *N*-phenyl and *N*-benzyl) give the highest γ-selectivity (>20 : 1). Oxidations of **6b–d** afford the corresponding chiral γ-hydroxy amides **7b–d** (≥94 : 6 er). CAHBs of **5c** and **5d** were carried out on gram scale giving chiral boronic esters **6c** (82%, 94.5 : 5.5) and **6d** (81%, 96.5 : 3.5 er) in good yield and without loss of enantioselectivity. CAHB/oxidation of an isomeric (*Z*)-alkene, benzyl amide (*Z*)-**5d**, affords results similar to those obtained with the (*E*)-isomer.

In contrast to amides **5a–d**, the analogous ester **8** is largely recovered unchanged upon attempted CAHB; only trace amounts of borylated products are identified along with some evidence for alkene isomerization. δ,ε-Unsaturated amide **9**, a one-carbon homologue of benzyl amide (*E*)-**5d**, is found to be considerably less reactive and less selective. Complete consumption of starting material requires 2% catalyst loading to afford 56% yield of a mixture of borylated products.^25^

Benzyl amides **5e–g** containing heteroaromatic ring systems are nonetheless good substrates under the standard CAHB/oxidation conditions yielding **7e–g**, respectively. Certain branched alkyl substituents (*i.e.*, **5h–j**) are also well tolerated. In particular, the chiral substrate (*E*)-**5j** demonstrates that (i) the proximal disubstituted alkene with respect to the amide directing group undergoes CAHB while the more distal trisubstituted alkene is untouched and (ii) the stereochemical course of γ-borylation is efficiently catalyst controlled. CAHB/oxidation with (*R*)-**L2** affords (4*S*,7*S*)-**7j**; (*S*)-**L2** affords (4*R*,7*S*)-**7j**. The silyl ether moiety in **5k** is tolerated and affords **7k** (78%, 97 : 3 er). Chiral acetal **5l** again undergoes catalyst controlled γ-borylation with high diastereoselectivity; (*R*)-**L1** affords (*S*,*S*)-**7l** (72%, 95 : 5 dr); (*S*)-**L1** affords (*R*,*S*)-**7l** in the same yield and diastereomer ratio. However, substrate **5m**, in which the chiral acetal moiety is in closer proximity to the site of hydroboration, shows a strong matched/mismatched effect. While (*R*)-**L1** affords (*R*,*S*)-**7m** (70%, 92 : 8 dr), the catalyst employing (*S*)-**L1** gives rise to a complex mixture of regioisomers **10**. Substrate **5n** (R^1^ = Me) also exhibits only modest regioselectivity (3 : 1), perhaps due to the size of the vinyl substituent compared to other derivatives described above; however, CAHB proceeds in good yield and high enantioselectivity (61%, 95 : 5 er).

**Fig. 3:**
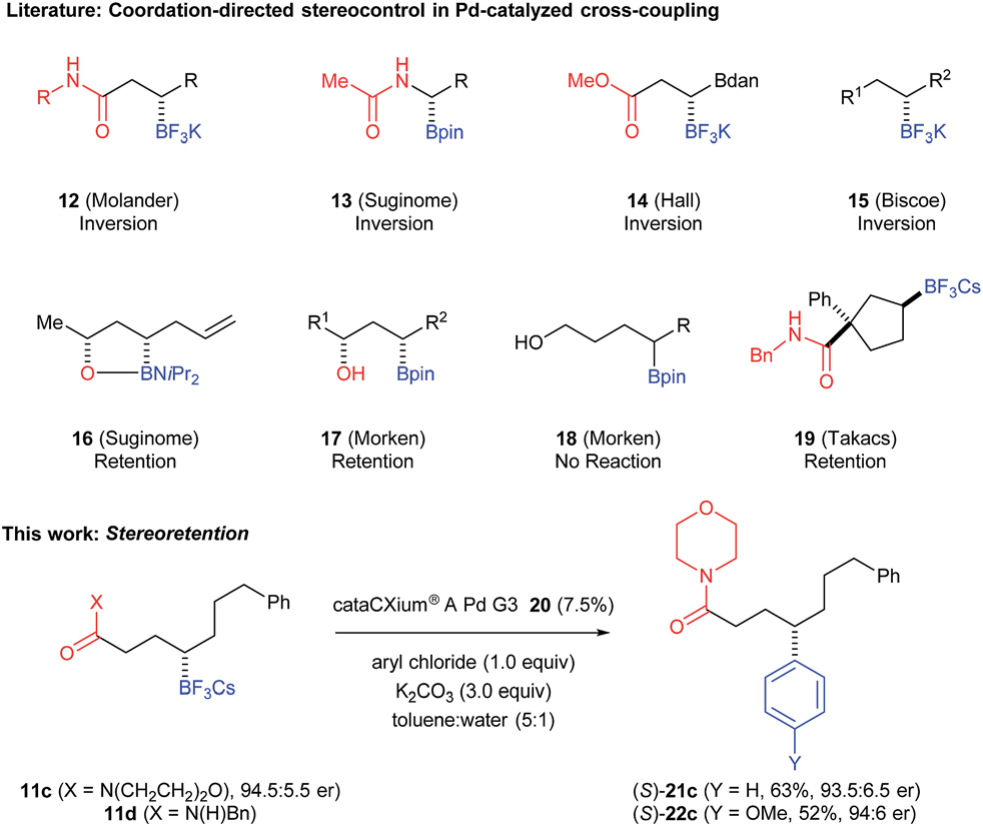
Stereochemical course of Suzuki–Miyaura cross-coupling with chiral secondary boronic esters or trifluoroborate salts.

Having developed an efficient method for the γ-borylation of γ,δ-unsaturated amides, Suzuki–Miyaura cross-coupling of **11** was examined (Fig. 3). Stereochemical aspects of the palladium-catalyzed cross-coupling of chiral secondary organoboron derivatives have recently attracted a great deal attention. Molander,^26^ Suginome,^27^ and Hall^28^ reported that β-borylated carbonyl derivatives **12–14**, whether as the boronic ester or the trifluoroborate salt, undergo cross-coupling with stereoinversion. The stereochemical course is rationalized by intramolecular coordination between the carbonyl oxygen and the boron atom of the boronic ester or the partially hydrolyzed trifluoroborate. The intramolecular coordination promotes invertive transmetallation resulting in overall stereoinversion for cross-coupling. Biscoe^19a,29^ also found stereoinversion for simple substrates lacking functionality needed for coordination to boron during the course of transmetallation (*e.g.*, **15**). On the other hand, Suginome^30^ reported that boracyclic intermediate **16** undergoes cross-coupling with stereoretention. Similarly, Morken^31^ reported that **17** undergoes hydroxyl-directed, inner-sphere, retentive transmetallation and overall cross-coupling with stereoretention. However, when the hydroxyl is one-carbon further removed, **18** fails to undergo cross-coupling under the otherwise same conditions. We have previously shown that **19** undergoes cross-coupling with overall stereoretention.^22^

Chiral boronic ester **6c** (*i.e.*, the morpholine amide) was converted to its corresponding trifluoroborate salt **11c** ^32^ and subjected to palladium-catalyzed cross-coupling using the Buchwald cataCXium® A Pd G3 (**20**) precatalyst.^22,33^ Cross-coupling with chlorobenzene yields amide (*S*)-**21c** (63%); 4-chloroanisole yields (*S*)-**22c** (52%). The products are obtained with essentially complete overall stereoretention.^34^ We find that the nature of the amide is important to the success of the cross-coupling. In contrast to the tertiary morpholine amide, the analogous secondary amide **11d** does not undergo cross-coupling under the conditions employed for **11c**. Hall *et al.* ^28*a*^ reported that β-boronic esters of secondary amides failed to cross-couple in cases where the corresponding tertiary amide coupled smoothly.

**Fig. 4:**
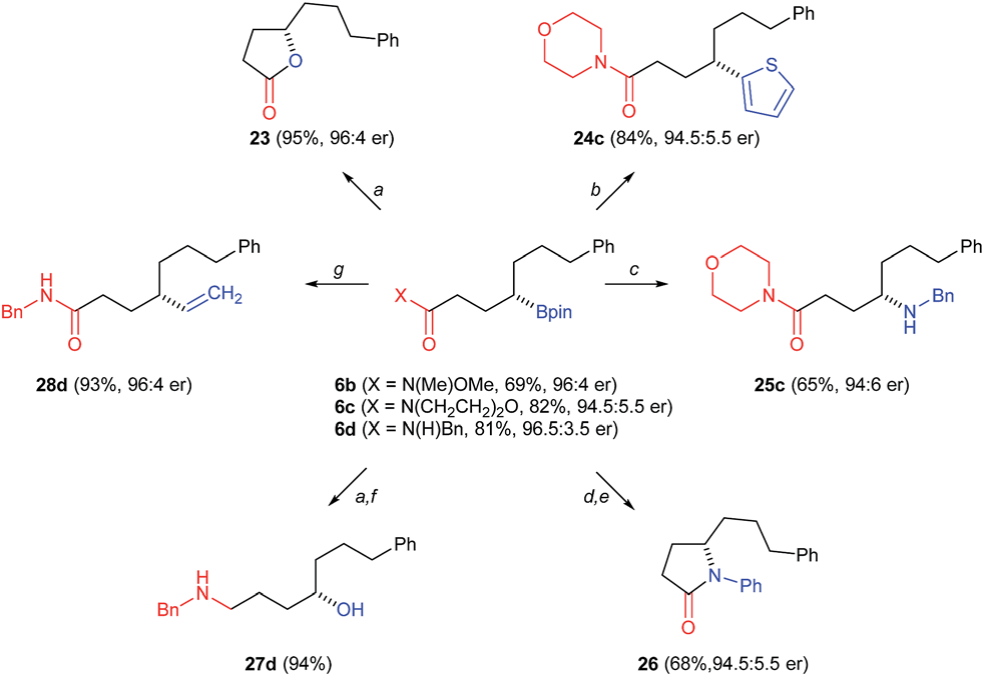
Stereospecific transformations of organoboranes **6b–d**. Conditions: (a) NaOH/H_2_O_2_; (b) (i) *n*-BuLi, thiophene, (ii) NBS; (c) BCl_3_, BnN_3_; (d) BCl_3_, PhN_3_; (e) 6 M HCl; (f) LiAlH_4_; (g) (i) vinylMgBr, (ii) I_2_/NaOMe.

Chiral organoboronates are useful for a variety of other stereospecific transformations. Fig. 4 illustrates several examples starting from chiral boronic esters **6b–d**; the latter are isolated in 69–82% yield from the corresponding alkenes. Treating **6b** with H_2_O_2_/aq. NaOH affords the known chiral 5-substituted-γ-lactone **23** (95%).^35^ As an alternative to palladium-catalyzed cross-coupling, the morpholine amide derivative **6c** undergoes stereoretentive cross-coupling with 2-lithiothiofuran under the conditions developed by Aggarwal^2*e*^ to give **24c** (84%). Compound **6c** also undergoes BCl_3_-assisted amination with benzyl azide under the conditions reported by Knochel^36^ to form the γ-amino acid derivative **25c** (65%). Phenyl azide also serves as a good nucleophile in such amination reactions, and **6c** is converted to the corresponding *N*-phenyl γ-amino acid en route to the 5-substituted-γ-lactam **26** (68%) by acid catalyzed cyclization. Benzyl amide derivative **6d** is efficiently converted to 1,4-aminoalcohol **27d** after oxidation of the C–B bond followed by amide reduction with LAH (94%). While the secondary *N*-benzyl amide **11d** failed in the attempted palladium-catalyzed cross-coupling described above, **6d** undergoes efficient vinylation in a sequence initiated the by treatment with excess vinyl Grignard;^2*h*^ amide **28d** is formed in high yield (93%).

## Conclusions

γ,δ-Unsaturated secondary (*i.e.*, *N*-phenyl and *N*-benzyl) and tertiary (*i.e.*, Weinreb and morpholine) amides undergo efficient rhodium-catalyzed CAHB to afford γ-borylated derivatives in good yield and with high levels of asymmetric induction; enantioselectivity as high as 97 : 3 er is observed. While two good alternative methods are available to prepare chiral secondary β-borylated carbonyl compounds, the present method of directed-CAHB provides to our knowledge the first direct route to chiral acyclic secondary γ-borylated carbonyl compounds with high regio- and enantioselectivity.

A previous study found β- and γ-borylation of related substrates differ in the sense of stereoinduction, *i.e.*, π-facial discrimination.^20*a*^ However, it is not the case in the present study; β-borylation of β,γ-unsaturated amide **1** and γ-borylation of the one-carbon homologue γ,δ-unsaturated amide **5** add to the same face of the alkene. In the present study, CAHB of a substrate bearing both di- and trisubstituted alkene moieties (*i.e.*, **5j**) occurs only on the disubstituted double bond proximal to the carbonyl group. Chiral substrates **5j** and **5l** undergo highly diastereoselective CAHB with catalyst control; however, substrate **5m**, in which the resident oxygen-bearing stereocenter resides adjacent to the alkene, exhibits a strong matched and mismatched effect with enantiomeric catalysts.

The γ-borylated products are found to undergo stereoretentive palladium-catalyzed Suzuki–Miyaura cross-coupling, presumably *via* amide-directed inner-sphere stereoretentive transmetallation, as well as stereoretentive C–B to C–C transformations using main group organometallic reagents (*e.g.*, lithium and magnesium). In addition, a variety of other stereospecific transformations are highlighted by the conversions of chiral, secondary γ-boronic esters **6b–d** to 1,4-amino alcohols, γ-amino acid derivatives, and 5-substituted-γ-lactone and γ-lactam ring systems. Further studies are in progress.
